# Supplementary figures and images for: Occult zonulopathy detected during cataract surgery in patients with acute primary angle closure: a retrospective study
Source: PeerJ. 2025 Apr 18;13:e19330. doi: 10.7717/peerj.19330 (PMC12010992; doi:10.7717/peerj.19330)

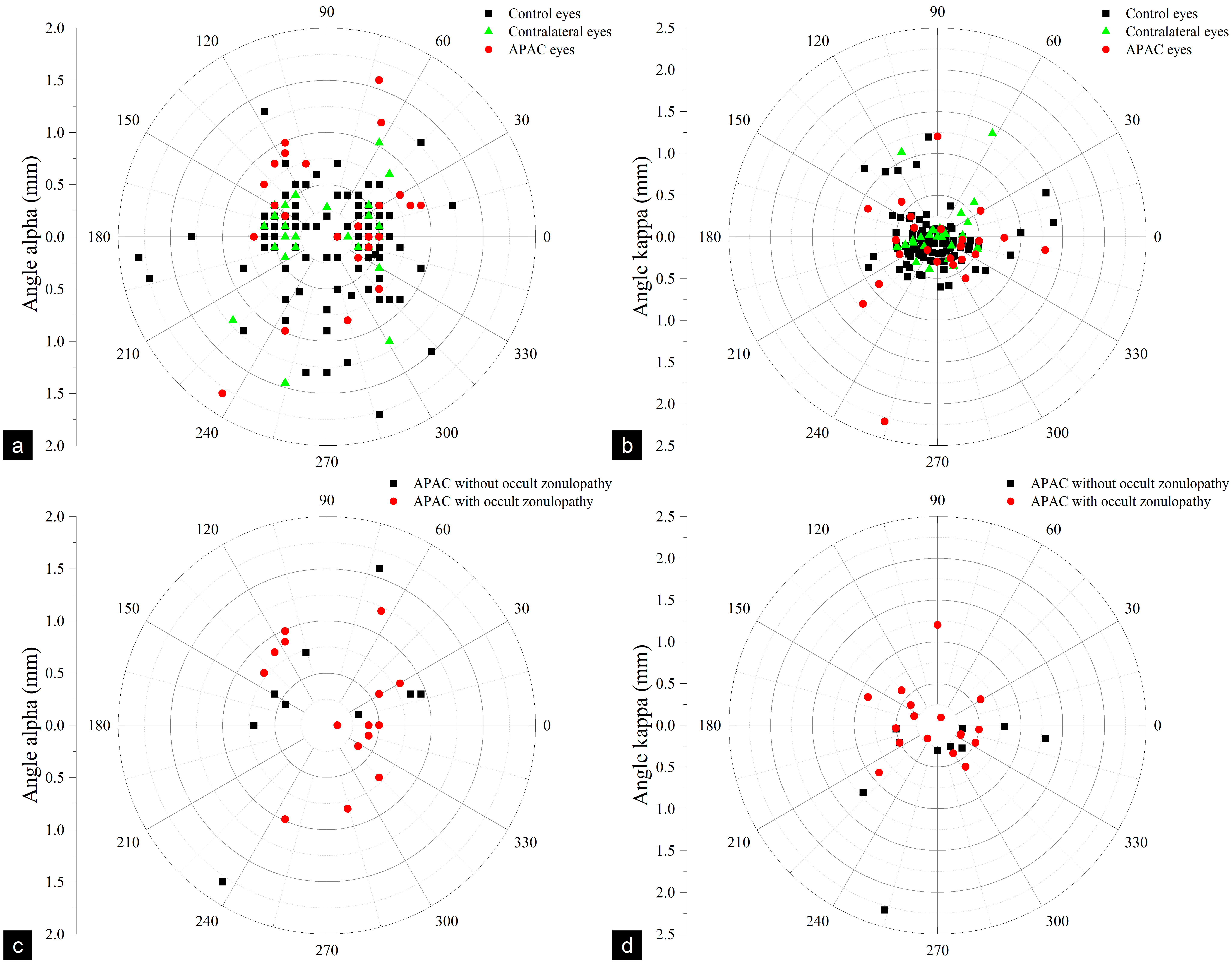

Supplement: Supplemental Information 3 — (A) APAC eyes showed a significantly greater angle alpha than control eyes (P = 0.012), but no difference compared to the contralateral eyes (P = 0.074). (B) APAC eyes had a significantly greater angle kappa than control eyes (P < 0.001) and the contralateral eyes (P = 0.011), but no difference between control eyes and the contralateral eyes (P = 0.713). (C–D) No significant difference in angle alpha and angle kappa was found in the APAC eye with and without occult zonulopathy (both P > 0.05). [file peerj-13-19330-s003.png]

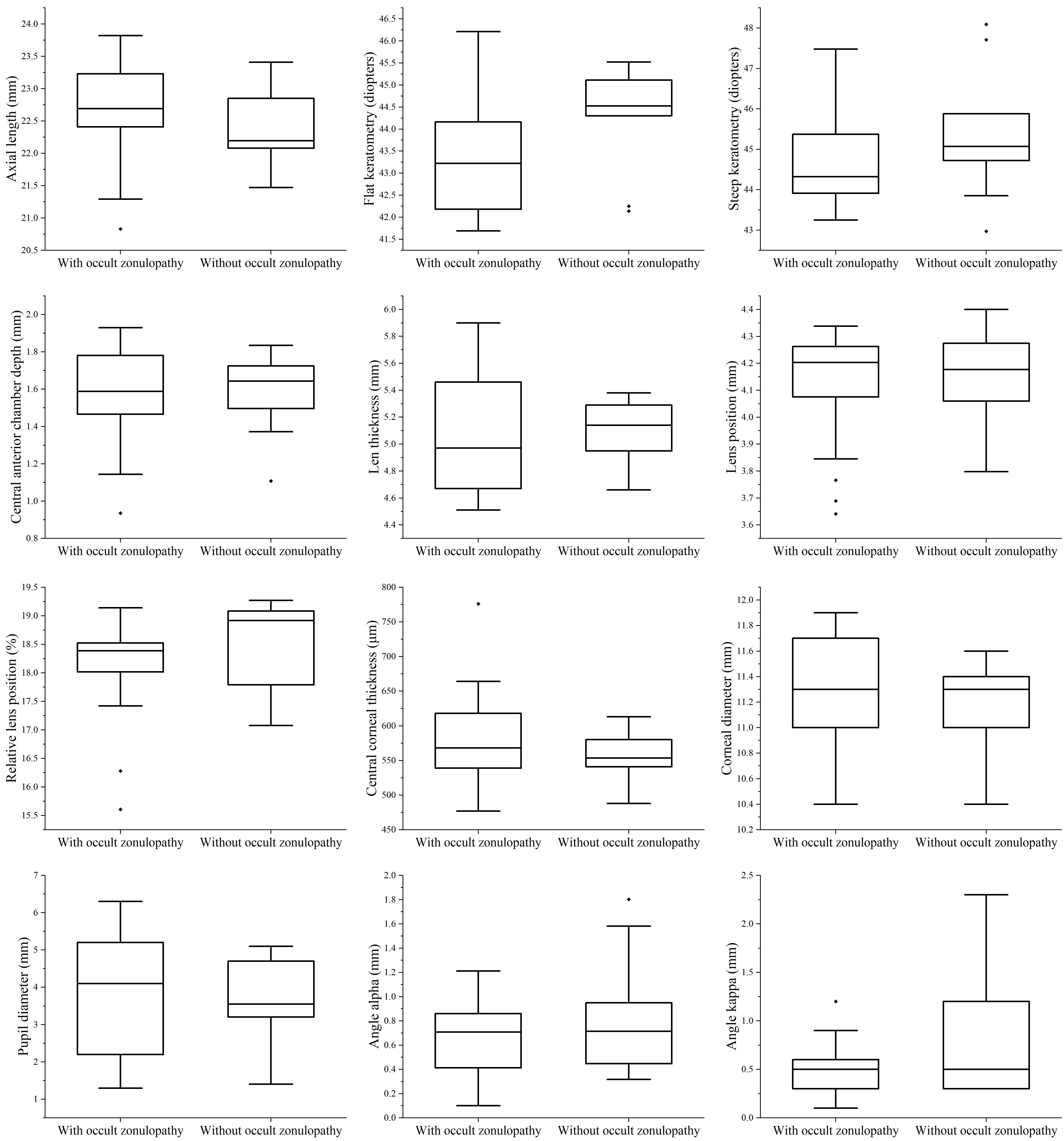

Supplement: Supplemental Information 4 — APAC eyes with occult zonulopathy showed longer AL, smaller keratometry, thinner LT, and RLP closer to anterior than that of APAC eyes without occult zonulopathy despite no statistical significance (all P > 0.05). [file peerj-13-19330-s004.png]
